# Characterization of clinical *enterococci* isolates, focusing on the vancomycin-resistant *enterococci* in a tertiary hospital in China: based on the data from 2013 to 2018

**DOI:** 10.1186/s12879-020-05078-4

**Published:** 2020-05-19

**Authors:** Wanqing Zhou, Hui Zhou, Yuhan Sun, Shuo Gao, Yan Zhang, Xiaoli Cao, Zhifeng Zhang, Han Shen, Chunni Zhang

**Affiliations:** 1 Department of Laboratory Medicine, Nanjing Drum Tower Hospital, The Affiliated Hospital of Nanjing University Medical School, 321# Zhongshan Road, Gulou District, Nanjing, Jiangsu Province 210008 P. R. China; 2 Department of Clinical Laboratory, Jinling Hospital, Nanjing University School of Medicine, Nanjing University, 305# East Zhongshan Road, Qinhuai District, Nanjing, Jiangsu Province 210008 P. R. China

**Keywords:** *Enterococcus*, VRE, *Enterococcus faecium*, *Enterococcus faecalis*, *vanA*, *vanM*

## Abstract

**Background:**

Vancomycin-resistant *Enterococcus* spp. (VRE) have spread all over the world. The present study aims to investigate the species distribution, specimen type and susceptibilities of *Enterococcal* species collected from Nanjing Drum Tower Hospital from 2013 to 2018. Additionally, distribution of VRE and prevalence of *van* gene among VRE isolates were also analyzed.

**Methods:**

The susceptibilities of 3913 *Enterococcus* isolates were retrospectively investigated. Among these strains, 60 VRE strains were further anazlyed in this study. The minimum inhibitory concentrations (MICs) of the VRE strains towards vancomycin, teicoplanin and linezolid were determined by E-test. Polymerase chain reaction (PCR) and DNA sequencing were used to investigate the prevalence of *van* genes among VRE. Furthermore, the sequence types (STs) of VRE strains were explored by multi-locus sequence typing (MLST).

**Results:**

Among the 3913 *enterococci* isolates, *Enterococcus faecalis* (*n* = 1870, 47.8%) and *Enterococcus faecium* (1738, 44.4%) were the main isolates. These *Enterococcus* strains were mainly isolated from urine (*n* = 1673, 42.8%), followed by secretions (*n* = 583, 14.9%) and ascites (*n* = 554, 14.2%). VRE displayed a decreasing trend year by year. Molecular analysis revealed that 49 out of 60 VRE isolates carried *vanA* gene, 10 carried *vanM*, and 1 carried both *vanA* and *vanM* genes. Sixteen distinct STs were identified among the 58 VREM, with ST78 (*n* = 16), ST192 (*n* = 8) and ST570 (*n* = 7) being the most dominant ones.

**Conclusions:**

*E. faecalis* and *E. faecium* were the major *enterococci* strains which are the main pathogens of urinary traction infections; *vanA* and *vanM* were the main determinants conferring resistance to vancomycin; ST78, ST192 and ST570 were the leading STs of VREM which displayed a decreasing trend of prevalence year by year.

## Background

*Enterococci* are leading pathogens of nosocomial infections such as bacteraemia, intra-abdominal infections and surgical wounds, especially urinary tract infections [1]. With the antimicrobial agents being frequently used in clinical treatment, antibiotic-resistant *enterococci*, particularly multi-drug resistant *enterococci* isolates, such as vancomycin-resistant *enterococci* (VRE) and linezolid-resistant *enterococci* (LRE) have emergence and spread all over the world [2, 3].

In Europe, an increasing proportion of vancomycin-resistant *E. faecium* (VREM) has been reported [4], with the VREM being increased from 11.2% in 2014 to 26.1% in 2017 in German hospitals [5]; In Canadian hospitals, the national prevalence of VRE tripled from 1.8% in 2007 to 6.0% in 2013 and peaked at 7.9% in 2011 [6]; Thus, besides high cost of treatment of VRE, high mortality and additional morbidity caused by the frequent incidence of infections has been reported in US and European countries [1]. Whereas in China, Antimicrobial Surveillance Network (CHINET) showed a declining trend of VRE year by year during 2013 to 2018 all over the China (http://www.chinets.com/Data/GermYear). However, infections of VRE have lead to increased cost and mortality compared to vancomycin-susceptible *Enterococcus* isolates [7]. The similar situation has also been observed on the distribution of VRE in Zhejiang, China during the period of 2015–2017 [8]. However, data on the characterization of VRE in tertiary hospitals in China are still limited.

To date, treatment options for infections caused by VRE are quite limited, including vancomycin, teicoplanin, linezolid and fosfomycin [1]. However, ranges on the MIC determinations of VRE on these antimicrobial agents are still unclear, which is very important for choice of drugs in clinical therapy.

Moreover, it has been well known that resistance to vancomycin in *Enterococci* is mediated by *van* genes. To date, *vanA*, *vanB*, *vanC*, *vanD*, *vanE*, *vanG*, *vanL*, *vanM*, and *vanN* have been identified [9]. *vanA* and *vanB* genotypes have predominated worldwide [10] and are mainly identified in VREM and vancomycin-resistant *E. faecalis* (VREF) isolates. Whereas, *vanM* gene was initially identified in a VREM clinical isolate in Shanghai in 2006 [11], subsequently reported in Singapore [12]. Presently, *vanM* has been the predominant gene in VRE in Shanghai since 2011 [13]. Noteworthiliy, albeit *vanM* gene has also been isolated in Hangzhou [14, 15], which is located approximately 200 km from Shanghai, and later in Beijing [16], it has not been detected in other cities in China. Thus, more data on the prevalence of VRE and the distribution of *van* genes among VRE in other cities of China are needed.

In this study, the strain types, sample sources, and susceptibilities of clinical *enterococci* collected in our hospital during 2013 to 2018 were retrospectively analyzed; Furthermore, the distribution of VRE strains, prevalence of *van* genes and sequence types (STs) among VRE were further investigated to analyze the characterization.

## Methods

### Bacterial strains

Consecutive and non-duplicate *Enterococcus* isolates were recovered in Nanjing Drum Tower Hospital (Nanjing, China) from January 2013 until December 2018 by WHONET 5.6 software. Confirmation of *enterococci* was performed using Vitek 2 Compact GP cards (bioMerieux, France). VRE were screened according to susceptibility to vancomycin (MIC > = 32 μg/ml). Where Antimicrobial testing was performed using Vitek 2 Compact GP67 cards (bioMerieux, France) or the disc diffusion method on Mueller-Hinton agar (bioMerieux, France), according to Clinical and Laboratory Standards Institute guidelines (CLSI, 2018) [17].

### Antimicrobial susceptibility testing

The minimum inhibitory concentrations (MICs) of VRE toward vancomycin, teicoplanin and linezolid were further determined by E-test (bioMerieux, France). The susceptibility of the VRE isolates to fosfomycin was tested by the disc diffusion method on Mueller-Hinton agar (bioMerieux, France). *E. faecalis* ATCC 29212 and *S. aureus* 29213 were used as quality controls in parallel. The antimicrobial susceptibility testing results were interpreted according to CLSI 2018 [17].

### DNA extraction and quantification

DNA extraction was performed by boiling method. Briefly, fresh colonies were picked into 1 ml sterile saline. After boiling for 10 min at 100 °C, followed by centrifugation for 30s with 12,000 r/sec, the supernatant was used to determine the DNA concentration and purity by using the NanoDrop 2000c spectrophotometer (Thermo Scientific, Waltham, MA, USA).

### Molecular detection of resistance genes

Amplification of *vanA*, *vanB*, *vanC1*, *vanC2/3*, *vanM*, and *vanD* was performed as previously described [11, 12]. Each batch of reactions contained a negative control, a positive control and a blank control. The amplicons were purified using a Qiagen DNA purification kit (Qiagen, Hilden, Germany) and subjected to sequencing. The sequence similarity was determined using the BLAST program from the National Center for Biotechnology Information (http://www.ncbi.nlm.nih.gov/BLAST).

### Multi-locus sequence typing (MLST)

MLST of *E. faecium* isolates was performed according to the method as previously described [18]. Briefly, the 7 housekeeping genes of *E. faecium* were amplified and sequenced, then strains were assigned to specific STs according to the protocol in MLST database (https://pubmlst.org/efaecium/).

### Statistical analysis

SPSS statistical software (version 20.0) was used to analyze the data. Difference on the resistance rates between the *E. faecalis* and *E. faecium* were analyzed by chi-square test. Chi-square values were corrected when the quantities of VRE isolates were less than 40. Statistical significance was defined when a *P*-value < 0.05.

## Results

### Distribution of clinical *enterococci* isolates from 2013 to 2018

Among the 3913 *enterococci* isolates, *E. faecalis* (*n* = 1870, 47.8%) and *E. faecium* (*n* = 1738, 44.4%) were the major ones, followed by *E. avium* (*n* = 169, 4.3%), *E. gallinarum* (*n* = 70, 1.8%), *E. casseliflavus* (*n* = 35, 0.9%), *E. durans* (*n* = 15, 0.4%), *E. cecorum* (*n* = 9, 0.2%) and *E. raffinosus* (*n* = 7, 0.2%). These *Enterococcus* were mainly isolated from urine specimens (*n* = 1673, 42.8%), followed by secretions (*n* = 583, 14.9%), ascites (*n* = 554, 14.2%), bile (*n* = 412, 10.5%), blood (*n* = 273, 6.9%), catheters (*n* = 169, 4.3%) and others (*n* = 249, 6.4%).

The annual distribution of *Enterococcus* isolates is shown in Table 1. It’s worthy to note, the proportion of ascite specimens increased annually, from 7.8% in 2013 to 21.8% in 2018, as shown in Table 2.

**Table 1:** Annual distribution of *Enterococci* isolates during 2013–2018

Year	*E. faecalis*	*E. faecium*	*E. avium*	*E.gallinarum*	*E.casseliflavus*	*E. durans*	*E. cecorum*	*E.raffinosus*	Total
2013	263	266	27	2	0	1	1	0	560
2014	316	286	17	7	3	2	1	0	632
2015	362	265	29	13	9	2	1	2	683
2016	305	276	32	17	3	4	3	4	644
2017	314	295	30	22	9	2	2	1	675
2018	310	350	34	9	11	4	1	0	719
Total	1870	1738	169	70	35	15	9	7	3913

**Table 2:** Samples distribution of *Enterococci* isolates from 2013 to 2018

Samples	2013		2014		2015		2016		2017		2018		Total	
	Num.	Ratio (%)	Num.	Ratio (%)	Num.	Ratio (%)	Num.	Ratio (%)	Num.	Ratio (%)	Num.	Ratio (%)	Num.	Ratio (%)
urine	256	45.7	284	44.9	308	45.1	266	41.3	267	39.6	292	40.6	1673	42.8
ascites	44	7.8	56	8.9	82	12	92	14.3	123	18.2	157	21.8	554	14.2
secretions	100	17.8	115	18.2	106	15.5	84	13	98	14.5	80	11.1	583	14.9
Bile	62	11.1	63	10	66	9.7	75	11.6	76	11.3	70	9.7	412	10.5
blood	34	6.1	38	6	41	6	51	7.9	45	6.7	64	8.9	273	6.9
catheter	25	4.5	31	4.9	30	4.4	25	3.9	28	4.1	30	4.2	169	4.3
others	39	7	45	7.1	50	7.3	51	8	38	5.6	26	3.7	249	6.4
Total	560		632		683		644		675		719		3913	100

### The susceptibilities of *E. faecalis* and *E. faecium*

**Fig. 1:**
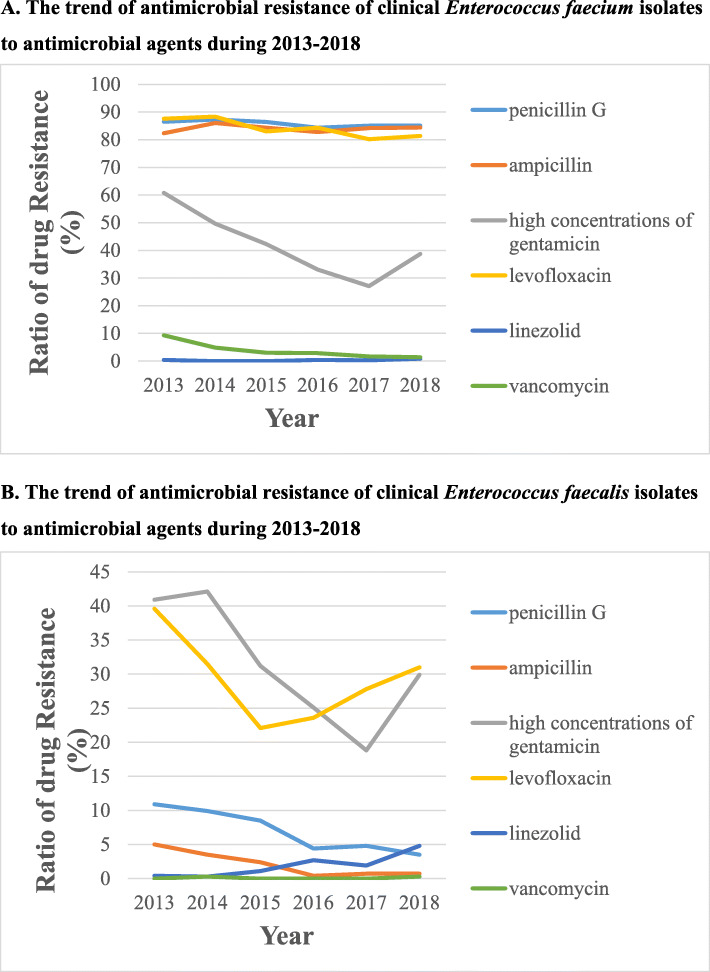
The trend of antimicrobial resistance of clinical enterococci isolates to antimicrobial agents during 2013–2018: **a** The trend of antimicrobial resistance of clinical *Enterococcus faecium* isolates to antimicrobial agents during 2013–2018; **b** The trend of antimicrobial resistance of clinical *Enterococcus faecalis* isolates to antimicrobial agents during 2013–2018

The resistance rates of *E. faecalis* and *E. faecium* were shown in Table 3. Overall, *E. faecium* showed obviously higher resistance rates than those of *E. faecalis* (*P* < 0.05)*.* For *E. faecium*, more than 40% of the isolates showed resistance toward penicillin G, ampicillin, high concentrations of gentamicin and levofloxacin***.*** Whereas, more than 95% of them displayed susceptibilities to linezolid, vancomycin, teicoplanin and tigecycline. In detail, from 2013 to 2018, high resistance rates to penicillin G, ampicillin and levofloxacin were shown to be above 80% every year; Resistance rates to high concentrations of gentamicin decreased yearly, from 60.8% in 2013 to 27.1% in 2017, but rebounded to 38.7% in 2018; Resistance rates to vancomycin decreased year by year, from 9.3% in 2013 to 1.4% in 2018 (Fig. 1a). Whereas, for *E. faecalis*, an increasing trend of resistance to linezolid was observed, from 0.4% in 2013 to 4.8% in 2018; In contrast, resistance rates to penicillin G and ampicillin decreased gradually; Resistance rates to high concentrations of gentamicin and levofloxacin decreased firstly and increased later (Fig. 1b).

**Table 3:** Resistance rates (%) of *E. faecalis* and *E. faecium* to antimicrobiol agents

Antibiotic	***E. faecalis*** (*n* = 1870)	***E. faecium*** (*n* = 1738)	*χ*^*2*^	*P* value
	Resistance rates (%)	Resistance rates (%)		
penicillin G	6.8	85.6	2146.5	< 0.05
ampicillin	2.1	83.8	2303.3	< 0.05
high concentrations of gentamicin	31	41.5	42.2	< 0.05
levofloxacin	28.2	84	1033.1	< 0.05
linezolid	1.9	0.4	16.3	< 0.05
vancomycin	0.1	3.7	65.5	< 0.05
teicoplanin	0	2.5	46.0	< 0.05
Tigecycline	0	0	…	…

Chi-square statistics was used to obtain *P* value

### Distribution and susceptibilities of VRE

**Fig. 2:**
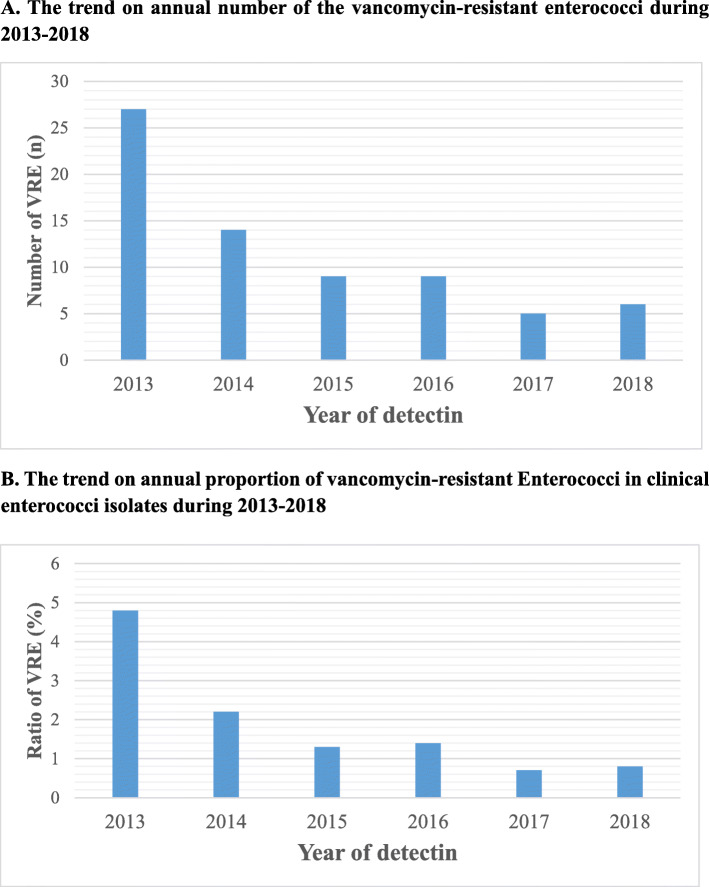
The trend on annual number/proportion of vancomycin-resistant Enterococci in clinical enterococci isolates during 2013–2018: **a** The trend on annual number of the vancomycin-resistant enterococci during 2013–2018; **b** The trend on annual proportion of vancomycin-resistant enterococci in clinical enterococci isolates during 2013–2018

Screening of VRE showed that 60 VRE, including 58 *E. faecium*, 1 *E. faecalis* and 1 *E. avium*, were included in this study. These VRE were recovered from urine (*n* = 31), catheter (*n* = 9), blood (*n* = 7), ascites (*n* = 6), wound secretions (*n* = 3), bile (*n* = 2), drainage fluid of perirenal abscess (*n* = 1) and hydrothorax (*n* = 1). And the distribution of VRE among every year during 2013–2018 presented a declining trend year by year (Fig. 2a, b).

Sixty VRE isolates showed high resistance to vancomycin (MIC > 256 μg/ml), and changeable resistance to teicoplanin (1 μg/ml ~ > 256 μg/ml). Fortunately, VRE strains showed 100% of susceptibility to linezolid (MIC ≤2 μg/ml) and to fosfomycin.

### The prevalence of *van* genes

All the 60 VRE strains carried at least one *van* gene. Among them, 49 strains carried *vanA* gene (47 *vanA* in *E. faecium*, 1 one in *E. faecalis* and 1 one in *E. avium*), 10 *E. faecium* carried *vanM* gene, and 1 *E. faecium* carried both *vanA* and *vanM*.

### The sequence types of VRE

Among the 58 VREM isolates, 16 distinct STs were identified, with ST78 (*n* = 16), ST192 (*n* = 8) and ST570 (*n* = 7) being the predominant ones, followed by ST203 (*n* = 6) and ST343 (*n* = 5). ST414 (*n* = 3), ST1015 (*n* = 2), ST17 (*n* = 2), ST789 (*n* = 2), ST1014 (*n* = 1), ST1039 (*n* = 1), ST18 (*n* = 1), ST202 (*n* = 1), ST323 (*n* = 1), ST564 (*n* = 1) and ST80 (*n* = 1) were also found. The distribution of the dominant clones of VRE in 2013–2018 were shown in Table 4. where ST 570 predominated in 2013 and 2015, whereas, ST203 and ST343 were mainly prevalent clones in 2013. Therefore, the epidemic strains spread to our hospital in 2013, and the prevalence of this major clone was scattered and presented in a variety of clonotypes.

**Table 4:** Distribution of *van* genes and sequence types among vancomycin-resistant *Enterococcus faecium*

Sequence types	Year						*van* genes			Total
	2013	2014	2015	2016	2017	2018	*vanA*	*vanM*	*vanA*/*vanM*	
17	0	2	0	0	0	0	2	0	0	2
18	1	0	0	0	0	0	1	0	0	1
78	6	4	2	1	1	2	15	1	0	16
80	0	1	0	0	0	0	1	0	0	1
192	1	4	1	0	2	0	3	4	1	8
202	0	0	1	0	0	0	1	0	0	1
203	5	1	0	0	0	0	5	1	0	6
323	0	0	0	0	0	1	1	0	0	1
343	5	0	0	0	0	0	5	0	0	5
414	2	1	0	0	0	0	2	1	0	3
564	0	0	0	1	0	0	0	1	0	1
570	3	0	3	1	0	0	7	0	0	7
789	0	0	0	1	1	0	1	1	0	2
1014	0	0	0	0	0	1	1	0	0	1
1015	1	0	0	0	1	0	2	0	0	2
1039	0	0	0	1	0	0	0	1	0	1
Total	24	13	7	5	5	4	47	10	1	58

## Discussion

The spread of VRE has been a global problem to public health. In this study, we provided data on the characterization including species and specimen distribution of clinical *enterococci* isolates, as well as prevalence of *van* genes among VRE in a tertiary hospital based on the data from 2013 to 2018.

We found that the resistance rates of *E. faecium* toward most of the antimicrobial agents in clinical treatment were higher than those of *E. faecalis*, especially to penicillin, ampicillin, and levofloxacin. This is in accordance with the previous report [8], providing evidence that *E. faecium* are prone to be more resistant than *E. faecalis*. Albeit that infections caused by *E. faecium* present a serious clinical challenge for physicians, and treatment options for these infections are limited [8]. Fortunately, good sensitivities toward vancomycin, linezolid, tigecycline and fosfomycin were displayed, which means that there are still potent effective drugs for the treatment of positive *cocci,* including *enterococci*.

The high distribution of VREM in our study is consistent with the previous report [19, 20], indicating that VREM is the major VRE. Moreover, in our study, VRE strains mainly originated from urine, which was different from the Canadian strains, where VREs primarily originated from blood (68.8%) [6]. Consistent with previous study (http://www.chinets.com/Data/GermYear), we observed a deceasing trend on the prevalence of VRE year by year, this may depend on the low distribution of VRE in Chinese hospitals [8]. Although it has been increasing steadily since the first description of these organisms in 2010 [21]. So far, the incidence of VRE infections has significantly increased, particularly in parts of Asia, Europe, and the United States [6, 22]. The situation in our study is totally different from the report in U.S. hospitals, where the number of U.S. hospitalizations with VRE discharges more than doubled between 2000 and 2006, with a prevalence as high as 65% [23]. Noteworthily, a weak upward trend (from 0.4% in 2013 to 2.4% in 2018) for linezolid-resistant *enterococci* was also observed in our study, together with the higher resistance rates of *E. faecalis* to linezolid than those of *E. faecium*, indicating that *E. faecalis* may be easy to develop resistance to linezolid than *E. faecium*, which alert us to carefully use linezolid in the clinical therapy [24]. Even though the high susceptibilities of VRE to linezolid in our study suggest that linezolid is a potentially effective for infections caused by VRE [3].

The high prevalence of *vanA* gene among VRE has been reported in several Asian countries, mainly South Korea, Japan, and China [25–27], which was also observed in our study. As we know that *vanA* type has been characterized by acquired resistance to high levels of both vancomycin and teicoplanin [9, 12], then we could provide explanation for the high resistance to vancomycin. Furthermore, a similar IS element, IS1216V, has been reported to be widespread among *vanA*-type VRE, and this element may play an important role in the dissemination of resistance determinants by transposon-mediated fusion of *vanA* plasmid with other plasmids [28, 29]. Thus, further study may be needed to investigate the distribution of such IS element to explore the probability of dissemination of *vanA* gene. As we know that, *vanM* was first identified in Shanghai in 2006 and later found to be the dominant gene mediating resistance to vancomycin in *E. faecium* [11]. Previous studies showed that *vanM* gene encodes a 343-amino-acid protein that shares 79.9% amino acid identity with *vanA* [11]; Phenotypically, *vanM*-type isolates showed similar antimicrobial susceptibility patterns to the *vanA* type, with resistance to both vancomycin and teicoplanin [11]. To date, *vanM* has been identified in Hangzhou [14, 15], Shanghai [11, 13] and Beijing [16] in China, but was not in other cities of China. In our study, the prevalence of *vanM* gene among VRE isolates suggests that this is the first time that we provided the related data in Nanjing, China. Furthermore, we found that all the *vanM*-containing isolates displayed resistance toward vancomycin, albeit the insertion of an IS1216-like element into the *vanM* gene cluster at various positions could lead to the silencing of the VanM phenotype by deletions or partial deletions of *vanR*, *vanS*, or *vanX* [15]. Additionally, *vanM*-containing vancomycin-susceptible *E. faecium* strains might switch to a vancomycin-resistant phenotype during prolonged vancomycin treatment, leading to a failure of vancomycin treatment [15]. Therefore, it may be necessary to strengthen the surveillance for *vanM* among clinical *Enterococcus* isolates [30]. More importantly, we also found a co-occurrence of *vanA* and *vanM* among a VREM, which has been reported previously [16]. To date, the co-existence of *vanA* and *vanC1* gene clusters in *E. gallinarum* isolates has been demonstrated to confer high-level glycopeptide resistance in southern India [31]; An outbreak due to *E. faecium* strain co-carrying *vanA* and *vanB* was reported in France [32]; In addition, *vanA*-VanD VRE strains were identified in Korea and France [33, 34]. Altogether, the co-occurrence of *van* genes among single strain indicated the importance of infection control measures.

MLST typing displayed 3 dominant STs, including ST78, ST192 and ST570, which were frequently identified in VREM isolates in China [13, 15, 35, 36]. This is quite different from the dominant clone of ST117 and ST80 in Germany [5]. As we know, ST78 has been reported to be the predominant ST among *vanA*- and *vanM*-type VREM strains in China [13, 16], although the dominant clone ST78-*vanA* as the most popular type in our study was mainly separated between 2013 and 2014 (Table 5); ST192 was the most prevalent clone for *vanM*-type VRE and *E. faecium* ST192 has been recognized as one of 3 highly prevalent STs responsible for hospital-associated (HA) bloodstream infections in German hospital patients [37]. Strain 4868 with ST192-*vanA*/*vanM* type detected in our study has also been reported in Beijing, China [16], which alerts us the probability of clone dissemination; In our study, ST570-*vanA* was mainly isolated in 2013 and 2015; ST343-*vanA* and ST203-*vanA* were detected only in 2013, and ST192-*vanM* was distributed in 2014 and 2017 (Table 5), these differences on the distribution of *van* genes in different clones annually may reflect the prevalent trend of major clone spread, which may relate with the differences in antibiotic use.

**Table 5:** Annual distribution of the combination of sequence types and *van* genotypes among vancomycin-resistant *Enterococcus faecium*

Types	2013 (*n* = 20)	2014 (*n* = 9)	2015 (*n* = 6)	2016 (*n* = 2)	2017 (*n* = 3)	2018 (*n* = 2)	Total
ST78-*vanA*	5	4	2	1	1	2	15
ST78-*vanM*	1	0	0	0	0	0	1
ST192-*vanA*	1	1	0	0	1	0	3
ST192-*vanM*	0	3	0	0	1	0	4
ST192-*vanA*/*vanM*	0	0	1	0	0	0	1
ST570-*vanA*	3	0	3	1	0	0	7
ST570-*vanM*	0	0	0	0	0	0	0
ST203-*vanA*	5	0	0	0	0	0	5
ST203-*vanM*	0	1	0	0	0	0	1
ST343-*vanA*	5	0	0	0	0	0	5
ST343-*vanM*	0	0	0	0	0	0	0

Our study has several limitations. Firstly, this is a retrospective study, not all the VRE isolates could be collected for the further analysis; Secondly, this is a single centre study, our data may not reflect all the characterization of VRE isolates from other institutions in China, since the burden of VRE has been shown to vary regionally.

## Conclusions

In conclusion, *E. faecalis* and *E. faecium* were the major *enterococci* strains which are the main pathogens of urinary traction infections. *vanA* and *vanM* were the main genes conferring resistance to vancomycin, ST78, ST192 and ST570 were the leading STs of VREM which displayed a decreasing trend year by year. Thus, our findings indicated the importance of performing regional antibiotic resistance surveillance for infection control practices.

## Data Availability

The datasets used and/or analysed during the current study are available from the corresponding author on reasonable request.

## Abbreviations

VRE: Vancomycin-resistant *enterococci*
VREM: Vancomycin-resistant *Enterococcus faecium*
VREF: Vancomycin-resistant *Enterococcus faecalis*
VREA: Vancomycin-resistant *E. avium*
MICs: Minimum inhibition concentrations
MLST: Multi-locus sequence typing
STs: Sequence types
